# Psychometric Properties of the French Version of the Spanish Burnout Inventory (SBI-FR) in Teachers

**DOI:** 10.3390/ejihpe15090182

**Published:** 2025-09-10

**Authors:** Ester Grau-Alberola, Hugo Figueiredo-Ferraz

**Affiliations:** 1 Facultad de Educación, Universidad Internacional de La Rioja (UNIR), 26006 Logroño, Spain; ester.grau@unir.net; 2 Unidad de Investigación Psicosocial de la Conducta Organizacional (UNIPSICO), Universitat de València, 46010 Valencia, Spain; 3 Facultad de Ciencias Sociales y Jurídicas, Universidad Internacional de Valencia (VIU), 46002 Valencia, Spain

**Keywords:** burnout, Spanish Burnout Inventory, teachers, validation, assessment

## Abstract

The Spanish Burnout Inventory (SBI) is one of the most widely used instruments for assessing burnout in Latin America, and several European countries. Based on a theoretical model, the SBI comprises 20 items distributed across four dimensions: Enthusiasm Toward the Job, Psychological Exhaustion, Indolence, and Guilt. Unlike traditional frameworks, the SBI incorporates guilt as a central factor, providing a more comprehensive understanding of burnout. The present study aimed to validate the French version of the instrument (SBI-FR) in a sample of 373 French teachers. Confirmatory factor analysis (CFA) supported the adequacy of the four-factor structure with satisfactory fit indices (RMSEA = 0.057, 90% CI, GFI = 0.911, NFI = 0.909, CFI = 0.948, AIC = 451.824). All items showed acceptable psychometric properties, and reliability coefficients were adequate across the four dimensions. These results support the SBI-FR as a valid and reliable tool for assessing burnout among teachers in France. Furthermore, the inclusion of guilt contributes to theoretical advances in the conceptualization of burnout, enabling the distinction between guilt-prone and non-guilt-prone profiles. Overall, this study offers empirical evidence for the cross-cultural applicability of the SBI and highlights its relevance as an alternative instrument for diagnosing burnout, particularly in educational settings.

## 1. Introduction

Work-related stress remains one of the most significant occupational health concerns in Europe, with nearly half of employees reporting it as a major problem. It is estimated that stress contributes to almost 50% of all workdays lost, which has serious consequences on productivity and public health (European Commission, 2021; EU-OSHA—European Agency for Safety and Health at Work, 2025). According to the World Health Organization (WHO, 2022), psychosocial risks in the workplace include ineffective communication and management practices, insufficient involvement in decision-making, low levels of autonomy, inadequate employee support, workload, inflexible schedules, and ambiguous organizational tasks. Such conditions are strongly associated with negative outcomes for both workers and organizations.

In France in particular, the deterioration of mental health in the workplace has become a growing concern. The Dares Analyses (Béatriz et al., 2021) showed that after the health crisis of 2020, the risk of depression among workers doubled, and perceived health declined sharply. Women, healthcare professionals, and employees in education and social services have been especially affected, as have executives and intermediate professions working remotely, all of whom have experienced intensified and deteriorated working conditions. These findings underscore the vulnerability of occupational groups whose work is both emotionally demanding and socially exposed (Vercambre et al., 2009).

Teachers represent one of the most affected professional categories. In their daily work, French teachers have to face objective difficulties—such as heavy workload, long commutes, tight schedules, and heterogeneity in students’ academic levels—as well as subjective difficulties, including student violence, classroom management challenges, and the expectations imposed by their institutions (Ciavaldani-Cartaut et al., 2017). Many teachers also report insufficient support from school administrators and a lack of recognition from society at large. Such conditions may leave them without adequate resources to cope effectively with their professional demands. According to national data, 11% of French teachers report high stress levels, particularly among younger teachers, placing them at the European average (Ministère de l’Éducation Nationale, de la Jeunesse et des Sports, 2020).

Against this backdrop, burnout emerges as a critical public health concern, defined as a psychological syndrome resulting from prolonged exposure to chronic interpersonal stressors at work (Maslach & Leiter, 2016) that are not successfully managed (World Health Organization [WHO], 2019/2021), with substantial implications for individual health and organizational functioning (Madigan et al., 2023). Gil-Monte (2005) conceptualizes the syndrome as a chronic response to work-related stress, particularly prevalent among professionals who provide services to others. In the educational sector, the combination of emotional demands, moral responsibilities, and institutional pressures has made teaching a high-risk profession for burnout (Figueiredo-Ferraz et al., 2021; Gil-LaOrden et al., 2024). Teacher burnout has become an increasingly significant concern during the COVID-19 pandemic (Fernández-Suárez et al., 2021; Weißenfels et al., 2022), driven by worries over unsafe school environments and the demands of remote teaching (Pressley et al., 2021).

Burnout may manifest in diverse forms, and empirical studies have identified distinct types or profiles of burnout (Boone et al., 2022; Guidetti et al., 2018; Holmström et al., 2023; Leiter & Maslach, 2016; Salmela-Aro & Upadyaya, 2020; Yaghmour et al., 2024). Consequently, research on burnout should evaluate the phenomenon considering the different profiles and their respective consequences. Such differentiation can be understood by recognizing that while some professionals exhibit pronounced clinical symptoms, personal suffering, diminished performance and increase substance use as burnout progresses, others may continue working within the organization for extended periods without significant personal distress, yet display patterns of detachment and indifference toward their work (Figueiredo-Ferraz et al., 2021; Gil-Monte, 2012; Misiolek-Marín et al., 2020; Olivares-Faúndez et al., 2014).

Gil-Monte’s Burnout Model (Gil-Monte, 2005) identifies two distinct worker profiles in the development of burnout. The first, referred to as Profile 1, includes individuals who employ distancing strategies to manage their disillusionment with work and psychological strain. These strategies often manifest as indolence and cynical attitudes toward clients, which ultimately degrade the quality of service and trigger client complaints. Nevertheless, these workers tend to feel at ease with the situation, as it allows them to remain in their positions for extended periods without experiencing significant health consequences. In contrast, Profile 2 describes workers for whom such coping mechanisms fail to alleviate work-related stress. These individuals experience guilt over the way they treat clients, a reaction that reflects the interpersonal nature of guilt. They perceive their behavior as a breach of the organization’s ethical standards, which can lead to adverse effects such as increased psychological distress, psychosomatic symptoms, and depression, ultimately resulting in medical leave rather than continued work engagement (Gil-Monte, 2005).

To address burnout effectively, the first step is accurate measurement. To measure it, researchers must operationalize the construct through psychometrically sound and contextually appropriate instruments. This transition from recognizing burnout as a problem to systematically measuring it is particularly important, which raises the need for reliable assessment tools (Villacura-Herrera et al., 2025).

The literature has identified several psychometric and conceptual limitations in the Maslach Burnout Inventory (MBI) (Maslach et al., 1997) as well as in other existing instruments for assessing burnout. For instance, some authors have argued that a two-factor structure may represent the data more adequately than the original three-factor model (De Beer & Bianchi, 2019; Lheureux et al., 2017) while others have highlighted problematic cross-loadings, particularly for Items 12 and 16 (Maslach et al., 1997). Furthermore, the Depersonalization subscale has frequently demonstrated low internal consistency (Figueiredo-Ferraz et al., 2013). Beyond these psychometric issues, the MBI has also been criticized for relying on a relatively narrow conceptualization of burnout (Halbesleben & Demerouti, 2005) and for having been originally conceived as a research tool rather than as a diagnostic instrument (Doulougeri et al., 2016).

To address these shortcomings, the Spanish Burnout Inventory (SBI) has been developed (Gil-Monte, 2019; Gil-Monte et al., 2023). The SBI evaluates the following dimensions: Psychological Exhaustion; Enthusiasm Toward the Job; Indolence, understood as indifferent or abusive behaviors toward clients; and Guilt.

Guilt is recognized as a key symptom of burnout (Gil-Monte, 2008; Maslach, 1982; Price & Murphy, 1984; Scully, 1983), and excessive or misdirected guilt may contribute to dysfunctional experience and clinical alterations. Some professionals may tend to underestimate contextual influences on behavior, attributing their actions to personality dysfunction. These feelings of guilt can create a vicious cycle. Guilt often leads to increased involvement, as the worker experiences remorse in an attempt to alleviate the guilt, perceives a personal failure, and feels compelled to take constructive action (Harder & Lewis, 1987).

The SBI has been validated across multiple European countries with different occupational samples (Alföldy & Gil-Monte, 2010; Bosle & Gil-Monte, 2010; Cramer et al., 2020; Figueiredo-Ferraz et al., 2013; Guidetti et al., 2018; Misiolek et al., 2017), and in different South America contexts (Carlotto et al., 2024; Deroncele-Acosta et al., 2023). These studies have consistently supported the factorial structure and reliability of the instrument, while underscoring the added theoretical value of incorporating guilt as a core dimension of burnout. However, until now there has not been a validated version of the SBI available for French-speaking teachers, despite the fact that this professional group has been identified as particularly vulnerable to burnout (Temam et al., 2019).

The present study addresses this gap. Its primary aim is to validate the French version of the Spanish Burnout Inventory (SBI-FR) among French teachers, by evaluating its factorial structure and psychometric properties. Specifically, we employ confirmatory factor analysis (CFA) to test the adequacy of the four-factor model, and we assess the reliability of its subscales through Cronbach’s alpha coefficients. By focusing on teachers, the study contributes to the development of culturally sensitive tools for assessing burnout in the French educational system. More broadly, the results have implications for advancing research on burnout, facilitating international comparisons, and improving diagnosis and intervention strategies in contexts where educational professionals face significant psychosocial risks.

## 2. Materials and Methods

### 2.1. Participants

A total of 377 questionnaires were received, of which 373 were retained for analysis. Four questionnaires were excluded due to incomplete responses.

In terms of sex distribution, 67 participants were men (18%) and 306 were women (82%). The mean age of the participants was 46.26 years (SD = 9.61), with ages ranging from 24 to 64 years. Regarding employment status, 91.2% of the participants held permanent contracts, while 8.8% were employed under temporary contracts. The average number of years of professional experience was 19 years (SD = 9.74).

Concerning the educational stage, 6.2% of the participants taught in Early Childhood Education, 48.8% in Primary Education, 39.7% in Secondary Education, and 5.4% in Higher Education.

### 2.2. Instruments

The Spanish Burnout Inventory (SBI; Gil-Monte, 2019) consists of 20 items distributed across four subscales: (1) Enthusiasm Toward the Job (5 items; e.g., “I see my job as a source of personal accomplishment”), which showed excellent internal consistency in the current sample (α = 0.92, 95% CI [0.907, 0.933]); (2) Psychological Exhaustion (4 items; e.g., “I feel emotionally exhausted”), with α = 0.88, 95% CI [0.851, 0.893]; (3) Indolence (6 items; e.g., “I think many students are unbearable”), with α = 0.78, 95% CI [0.740, 0.810]; and (4) Guilt (5 items; e.g., “I regret some of my behaviors at work”), with α = 0.85, 95% CI [0.820, 0.870]. Responses are provided on a five-point Likert-type scale ranging from 0 (Never) to 4 (Very frequently: Every day). High levels of burnout are reflected by low scores on the Enthusiasm Toward the Job subscale, in combination with elevated scores on Psychological Exhaustion, Indolence, and Guilt.

### 2.3. Procedure

This cross-sectional study was carried out among French teachers, who were invited to complete a questionnaire. Participant recruitment was based on snowball sampling, a non-probabilistic method. Participants received detailed information regarding the research objectives and procedures and were informed that their participation was entirely voluntary. Recruitment was carried out through disinterested, anonymous, and voluntary collaboration of French teachers via major platforms such as LinkedIn and French teaching groups on Facebook. Data were collected using Google Forms, an online survey tool that facilitated efficient distribution and data management. The data collection period spanned from November 2021 to January 2022.

To develop the French version, the methodological criteria of the International Test Commission (ITC) for the adequate adaptation of instruments to other cultures were followed, as explained in the manuscript (Muñiz & Hambleton, 2000). Specifically, the items of the SBI scale were translated from Spanish into French by a bilingual academic and approved by two experts in the fields of burnout and psychometrics. Subsequently, the SBI was back-translated by another bilingual academic different from the first. The two versions obtained were compared, discussed, and revised until full agreement was reached among the translators and experts. Semantic equivalence demonstrated satisfactory results, with all items achieving between 95% and 100% agreement.

### 2.4. Data Analysis

The data were analyzed using Confirmatory Factor Analysis (CFA) conducted with the Amos 20 software, applying the maximum likelihood estimation method. Given the sensitivity of the chi-square (χ^2^) test to sample size, additional fit indices were considered.

Data analysis was conducted in three stages. First, an item analysis was performed. Second, the factor structure of the SBI scores was examined through confirmatory factor analysis (CFA). Lastly, the reliability of the SBI subscale scores was evaluated.

The Goodness of Fit Index (GFI) reflects the proportion of variance and covariance accounted for by the model. Similarly, the Normed Fit Index (NFI) and the Comparative Fit Index (CFI) assess the extent to which a proposed model improves upon a baseline model in terms of explaining variance and covariance. For all three indices, values above 0.90 are generally regarded as indicative of a satisfactory model fit (Bentler, 1992; Hoyle, 1995).

The Root Mean Square Error of Approximation (RMSEA) provides an estimate of the model’s overall error. RMSEA values ranging from 0.05 to 0.08 suggest an acceptable level of fit (Browne & Cudeck, 1993; Hair et al., 1995).

Model comparisons were further supported by the Akaike Information Criterion (AIC), with preference given to the model showing the lowest AIC value, as it is considered to best represent the data (Akaike, 1987). As a general guideline, a difference of more than 4 in AIC values is seen as strong evidence in favor of the model with the smaller AIC (Burnham & Anderson, 2002).

## 3. Results

### 3.1. Item Analysis

Descriptive statistics for individual items are presented in Table 1. The highest mean score was observed for Item 8, belonging to the Psychological Exhaustion scale (M = 2.76) (“I thought that I was saturated by my work”). Conversely, the lowest mean score was recorded for Item 11 (M = 0.64) (“I want to be ironic with some students”), which is part of the Indolence scale.

Regarding the Enthusiasm towards the Job subscale, the highest mean was observed for Item 1 (M = 2.68) (“I find my work is a stimulating challenge”), while the lowest mean was recorded for Item 15 (M = 2.21) (“I find my work quite rewarding”).

For the Psychological Exhaustion subscale, Item 8 yielded the highest mean (M = 2.76) (“I feel I am overwhelmed by work”), whereas Item 12 displayed the lowest mean (M = 2.09) (“I feel weighed down by my job”).

With respect to the Indolence subscale, the highest mean was associated with Item 3 (M = 1.79) (“I think many students are unbearable”), and the lowest mean was found for Item 11 (M = 0.64) (“I feel like being sarcastic with some students”).

As for the Guilt subscale, Item 9 reported the highest mean (M = 1.41) (“I feel guilty about some of my attitudes at work”), while Item 16 exhibited the lowest mean (M = 0.84) (“I think I should apologize to someone for my behavior at work”).

All items showed corrected item-total correlations exceeding r = 0.40, indicating satisfactory item discrimination. Moreover, each item contributed positively to the internal consistency of its respective subscale.

In terms of distribution, items from the Enthusiasm Toward the Job and Psychological Exhaustion subscales exhibited negative skewness, suggesting a tendency for responses to cluster at the higher end of the scale. Conversely, items from the remaining subscales showed a pattern of positive skewness. Importantly, none of the 20 items exceeded the acceptable skewness threshold of ±2, supporting the assumption of approximate normality in item distributions.

### 3.2. Factor Analysis

Table 2 displays the data fit results for the SBI models. The four-factor model (M_4_) obtained the best data fit for the sample: *X*^2^(164) = 359.824, *p* < 0.001, RMSEA = 0.057, 90% confidence intervals (CIs) [0.049, 0.065], GFI = 0.911, NFI = 0.909, CFI = 0.948, and AIC = 451.824.

**Figure 1:**
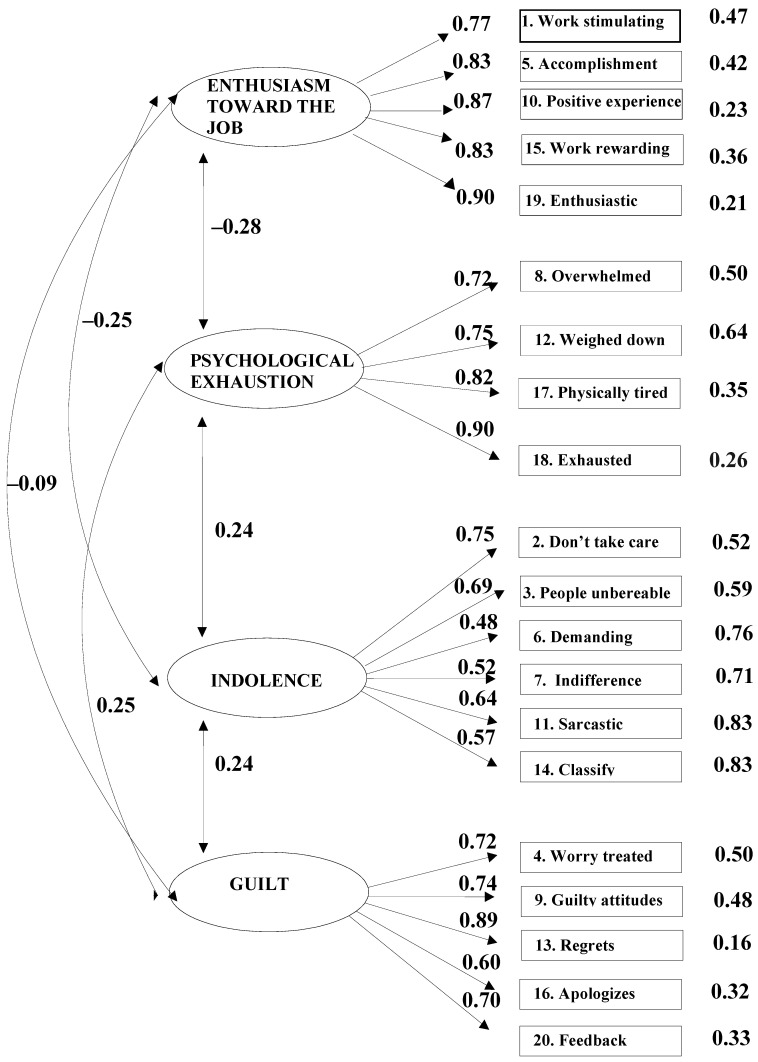
Results of the factorial model of the French version of the Spanish Burnout Inventory for teachers.

All factor loadings were statistically significant, and all interrelations among the SBI dimensions reached significance at *p* < 0.001 (see Figure 1). Moreover, chi-square difference tests revealed statistically significant results for all model comparisons, indicating that Model 4 (M_4_) provided a significantly better fit to the data compared to the alternative models (i.e., M_1_ to M_3_). Values of the difference in chi-square were as follows: M_1_ versus M_2_: *X*^2^(1) = 451.17, *p* = 0.001; M_2_ versus M_3_: *X*^2^(2) = 690.555, *p* = 0.001; and M_3_ versus M_4_: *X*^2^(3) = 390.161, *p* = 0.001. Considering the AIC index, Model 4 (M_4_) yielded the lowest AIC value. The difference in AIC between Model 3 (M_3_) and Model 4 (ΔAIC = 384.161) exceeded the threshold of 4, indicating a substantially better fit for M_4_.

All item-factor loadings were statistically significant. The lowest standardized loading was observed between Item 6 (“I think the relatives of students are very demanding”) and the Indolence factor, with a parameter estimate of 0.48 (see Figure 1).

Examination of the modification indices did not reveal any indications of cross-loadings. Furthermore, allowing any item to load on an alternative dimension would not have improved the overall model fit.

### 3.3. Validity of the Sub-Scales

Table 3 presents the descriptive statistics for the SBI subscales. Skewness and kurtosis values for all four subscales fell within the acceptable range of ±1, suggesting that the data approximate a normal distribution. The internal consistency of each subscale exceeded the widely accepted criterion of Cronbach’s alpha > 0.70, as proposed by Nunnally (1978). Furthermore, each item demonstrated a positive contribution to the overall reliability of its corresponding subscale, thereby reinforcing their internal coherence (Table 1). All four SBI dimensions exhibited significant intercorrelations. The strongest association was observed between Enthusiasm towards the job and Psychological exhaustion (r = −0.41, *p* < 0.001), while the weakest correlation emerged between Enthusiasm towards the job and Guilt (r = −0.12, *p* < 0.01) (Table 3).

## 4. Discussion

The aim of this paper was to establish evidence for the psychometric properties of SBI among French teachers.

The item-level results were consistent with theoretical expectations. Given the sample size, skewness and kurtosis values for all items were within acceptable limits. Although Items 16 and 20 from the Guilt subscale exhibited kurtosis values slightly exceeding the commonly accepted ±1 threshold for normality assessment, neither value surpassed ±2. Thus, the deviation from normality can be considered minimal (Miles & Shevlin, 2005).

The findings supported the hypothesized four-factor structure, consistent with the original Spanish version (Gil-Monte et al., 2006). Accordingly, it can be concluded that the factorial structure adequately replicates the theoretical model of the SBI, which conceptualizes burnout across four dimensions: Enthusiasm towards the Job, Psychological Exhaustion, Indolence, and Guilt.

The model’s fit indices were favourable: the GFI exceeded 0.90, and the CFI and NFI reached 0.95 and 0.91, respectively. Based on the residuals, the model demonstrated an adequate fit, as the RMSEA value was below 0.08 (Browne & Cudeck, 1993; Hair et al., 1995), and indeed fell beneath the more stringent threshold of 0.06 recommended by Hu and Bentler (1999). These findings are analogous to those observed in samples of professionals in other language in different nations: Portuguese teachers and nurses (Figueiredo-Ferraz et al., 2013), Italy nursing staff (Viotti et al., 2015), Poland anaesthesiologists (Misiolek et al., 2017) Chilean service professionals (Olivares-Faúndez et al., 2018), Colombian surgical specialist (García et al., 2022) and Valencian/Catalonian speaking non-university teachers (Llorca-Rubio et al., 2021). This structure reinforces the conceptual framework of the four burnout symptoms: Enthusiasm towards the job, Psychological exhaustion, Indolence, and Guilt.

The results also indicate that the items possess suitable psychometric properties relative to their respective subscales. The corrected item-scale correlation values are robust, suggesting that each dimension of the SBI may be regarded as a linear function of its constituent items. Item 06 (“I think the relatives of students are very demanding”) displayed the lowest moderate correlation with its factor by a noticeable margin. A comparable observation was made in studies conducted in Mexico (Mercado-Salas & Gil-Monte, 2012), among high school teachers (N = 505), and in Italy (Guidetti et al., 2018) involving 689 teachers. In these investigations, item 06 again yielded the lowest factor loading (λ = 0.47; λ = 0.44)

All four subscales demonstrated satisfactory internal consistency, with Cronbach’s alpha values ranging from 0.78 to 0.92. The subscales of Enthusiasm towards the job, Psychological exhaustion, and Guilt surpassed the stringent 0.80 criterion (Henson, 2001), and the Cronbach’s alpha for Indolence exceeded 0.70 (Nunnally, 1978). These results are consistent with those obtained in on a previous study carried out with sample from Italy (Guidetti et al., 2018) among elementary and middle school teachers, in Portugal in a study conducted with teachers and nurses (Figueiredo-Ferraz et al., 2013) and improves on a previous Spanish study with a smaller sample of employees working with intellectually disabled individuals, which reported a Cronbach’s alpha of 0.66 for Indolence (Gil-Monte et al., 2005).

Considering the sample size, we can conclude that these findings are stable among French teachers. They are also consistent with previous research in Italy, Brazil, Chile, Mexico, Spain and Portugal. Nonetheless, further studies are required to determine whether similar outcomes would emerge in other professional contexts and countries.

## 5. Conclusions

The significance of the present study lies in its provision of empirical evidence supporting the psychometric adequacy of an alternative measure of burnout. In the effort to advance burnout research, it is essential for researchers and practitioners to employ instruments that not only exhibit robust psychometric properties but also offer a broader conceptualization of burnout than traditional measures. The SBI is a well-established and psychometrically sound instrument suitable for use in international studies and surveys addressing burnout, job-related stress outcomes, and overall quality of working life.

The SBI introduces a theoretical framework aimed at elucidating distinct manifestations of burnout. Its contribution to the field rests in providing an expanded perspective on the syndrome, thereby facilitating the diagnosis and treatment of individuals affected by burnout. In clinical practice, the early identification of burnout symptoms may contribute to mitigating symptom progression and expediting recovery. Notably, the SBI emphasizes the assessment of guilt as a core symptom of burnout, thereby enabling a more comprehensive diagnosis, distinguishing among affected individuals, and recognizing its potential impact on health disorders (Gil-Monte, 2019; Gil-Monte et al., 2023).

Despite the strengths of this study, it is important to recognize certain limitations. One limitation concerns the use of self-reported data, which entails an inherent risk of response and recall bias. Another limitation is the gender imbalance among participants (18% of participants were men). It is recommended that comparative studies be conducted, considering key sociodemographic variables known to affect burnout, such as gender, type of work, and related factors. The study’s findings should be interpreted with caution, as their applicability to the entire population of French teachers is constrained by the predominance of primary and secondary education teachers in the sample.

Future research should aim, on the one hand, to expand the sample across different educational levels in France in order to enhance the generalizability of the results, and, on the other hand, to conduct a cross-validation of the model and establish clinical cut-off scores based on the SBI—similar to those already developed for Spain and Portugal—so as to accurately assess the true epidemiological burden of burnout in the French context.

## Figures and Tables

**Table 1:** Descriptive statistics of Spanish Burnout Inventory—French version (SBI-FR) items.

Subscale Item	Mean (SD)	Corrected Item-Scale Correlations	Skewness	Kurtosis	Alpha If Item Deleted
Enthusiasm Toward the Job (α = 0.92)	2.39 (0.93)		−0.32	−0.49	
1	2.68 (1.07)	0.74	−0.48	−0.56	0.91
5	2.40 (1.16)	0.81	−0.28	−0.81	0.90
10	2.44 (0.99)	0.82	−0.26	−0.51	0.90
15	2.21 (1.06)	0.77	−0.06	−0.61	0.91
19	2.24 (1.04)	0.85	−0.18	−0.73	0.89
Psychological Exhaustion (α = 0.88)	2.49 (0.95)		−0.27	−0.69	
8	2.76 (1.03)	0.68	−0.49	−0.40	0.86
12	2.09 (1.21)	0.68	−0.03	−0.92	0.86
17	2.63 (1.05)	0.76	−0.35	−0.52	0.83
18	2.47 (1.16)	0.80	−0.27	−0.88	0.81
Indolence (α = 0.78)	1.56 (0.74)		0.51	0.29	
2	1.72 (1.08)	0.63	0.26	−0.65	0.71
3	1.79 (1.06)	0.59	0.35	−0.57	0.73
6	1.83 (0.99)	0.41	0.37	−0.31	0.77
7	1.03 (0.99)	0.46	0.93	0.66	0.76
11	0.64 (1.19)	0.56	0.27	−0.79	0.73
14	1.36 (1.11)	0.49	0.67	−0.15	0.75
Guilt (α = 0.85)	1.14 (0.71)		0.67	0.58	
4	1.27 (1.01)	0.67	0.77	0.25	0.81
9	1.41 (1.03)	0.66	0.59	0.00	0.82
13	1.22 (0.90)	0.79	0.61	0.29	0.78
16	0.84 (0.70)	0.56	0.75	1.27	0.84
20	0.98 (0.80)	0.64	0.85	1.29	0.82

**Table 2:** Model fit for the Spanish Burnout Inventory in French teachers.

Model	*X* ^2^	*df*	RMSEA_(90% CI)_	GFI	NFI	CFI	AIC
M_1_ (1 factor)	1891.714	170	0.179 [0.172, 0.187]	0.499	0.459	0.480	1971.714
M_2_ (2 factors)	1440.544	169	0.142 [0.135, 0.149]	0.641	0.637	0.663	1522.544
M_3_ (3 factors)	749.985	167	0.097 [0.090, 0.104]	0.788	0.811	0.846	835.985
M_4_ (4 factors)	359.824	164	0.057 [0.049, 0.065]	0.911	0.909	0.948	451.824

*Note*_1_. *df* = degrees of freedom; RMSEA_(CI)_ = Root Mean Square Error of Approximation; GFI = Goodness of Fit Index; NFI = Normed Fit Index; CFI = Comparative Fit Index; AIC = Akaike Information Criterion. *Note*_2_. For all chi-square values, *p* ≤ 001.

**Table 3:** Descriptive statistics for SBI-FR dimensions, and correlations between dimensions.

	Sk	Ku	Range	1	2	3	4
1. Enthusiasm towards job	−0.32	−0.49	0–4				
2. Psychological Exhaustion	0.27	−0.69	0–4	−0.41 **			
3. Indolence	0.51	0.29	0–4	−0.33 **	0.32 **		
4. Guilt	0.67	0.58	0–4	−0.12 *	0.40 **	0.34 **	

** *p* < 0.001; * *p* < 0.01.

## Data Availability

The raw data supporting the conclusions of this article will be made available by the authors upon request.
